# A chronological review of COVID-19 case fatality rate and its secular trend and investigation of all-cause mortality and hospitalization during the Delta and Omicron waves in the United States: a retrospective cohort study

**DOI:** 10.3389/fpubh.2023.1143650

**Published:** 2023-09-15

**Authors:** Jing-Xing Li, Pei-Lun Liao, James Cheng-Chung Wei, Shu-Bai Hsu, Chih-Jung Yeh

**Affiliations:** ^1^Department of Internal Medicine, Taipei Veterans General Hospital, Taipei, Taiwan; ^2^School of Medicine, China Medical University, Taichung, Taiwan; ^3^Graduate Institute of Clinical Laboratory Sciences and Medical Biotechnology, National Taiwan University, Taipei, Taiwan; ^4^Department of Medical Research, Chung Shan Medical University Hospital, Taichung, Taiwan; ^5^Department of Nursing, Chung Shan Medical University, Taichung, Taiwan; ^6^Institute of Medicine, Chung Shan Medical University, Taichung, Taiwan; ^7^Department of Allergy, Immunology & Rheumatology, Chung Shan Medical University Hospital, Taichung, Taiwan; ^8^Graduate Institute of Integrated Medicine, China Medical University, Taichung, Taiwan; ^9^College of Medicine, China Medical University, Taichung, Taiwan; ^10^Department of Nursing, China Medical University Hospital, Taichung, Taiwan; ^11^Department of Public Health, Chung Shan Medical University, Taichung, Taiwan

**Keywords:** coronavirus disease 2019, COVID-19, case fatality rate, CFR, mortality trends, SARS-CoV-2

## Abstract

**Introduction:**

Coronavirus disease 2019 (COVID-19) has caused more than 690 million deaths worldwide. Different results concerning the death rates of the Delta and Omicron variants have been recorded. We aimed to assess the secular trend of case fatality rate (CFR), identify risk factors associated with mortality following COVID-19 diagnosis, and investigate the risks of mortality and hospitalization during Delta and Omicron waves in the United States.

**Methods:**

This study assessed 2,857,925 individuals diagnosed with COVID-19 in the United States from January 2020, to June 2022. The inclusion criterion was the presence of COVID-19 diagnostic codes in electronic medical record or a positive laboratory test of the SARS-CoV-2. Statistical analysis was bifurcated into two components, longitudinal analysis and comparative analysis. To assess the discrepancies in hospitalization and mortality rates for COVID-19, we identified the prevailing periods for the Delta and Omicron variants.

**Results:**

Longitudinal analysis demonstrated four sharp surges in the number of deaths and CFR. The CFR was persistently higher in males and older age. The CFR of Black and White remained higher than Asians since January 2022. In comparative analysis, the adjusted hazard ratios for all-cause mortality and hospitalization were higher in Delta wave compared to the Omicron wave. Risk of all-cause mortality was found to be greater 14–30 days after a COVID-19 diagnosis, while the likelihood of hospitalization was higher in the first 14 days following a COVID-19 diagnosis in Delta wave compared with Omicron wave. Kaplan–Meier analysis revealed the cumulative probability of mortality was approximately 2-fold on day 30 in Delta than in Omicron cases (log-rank *p* < 0.001). The mortality risk ratio between the Delta and Omicron variants was 1.671 (95% Cl 1.615–1.729, log-rank *p* < 0.001). Delta also had a significantly increased mortality risk over Omicron in all age groups. The CFR of people aged above 80 years was extremely high as 17.33%.

**Conclusion:**

Male sex and age seemed to be strong and independent risk factors of mortality in COVID-19. The Delta variant appears to cause more hospitalization and death than the Omicron variant.

## 1. Introduction

Coronavirus disease 2019 (COVID-19) is an ongoing pandemic caused by severe acute respiratory syndrome coronavirus 2 (SARS-CoV-2). COVID-19 can cause various complications that can be fatal. The most prevalent complications include pneumonia, respiratory failure, myocardial injury, renal failure, sepsis or complications related to thrombosis (1). There have also been reports of individuals experiencing a flare-up of autoimmune diseases following infection with SARS-CoV-2 (1, 2). Over time, various mutated variants of COVID-19 have emerged and been identified. With regards to the Alpha variant, studies suggest that the absolute risk of death within 28 days of infection increases with age and the presence of comorbidities. Furthermore, this variant appears to spread more rapidly and have a higher mortality rate than the pandemic strain seen prior to its emergence (3).

In early 2022, daily newly confirmed cases remarkably rose, attributed to a novel variant called Omicron (lineage BA.1.1), which invaded the United States and became the dominant variant at the end of December 2021 (4, 5). Omicron was more infectious and transmissible with immune escape than the wild-type strain and the 4 other variants of concern (VOC) (6). It was thought that the clinical severity of infection is lower for Omicron than for Delta (7–13). All literature described lower mortality of Omicron variant than Delta variant. According to data from the CDC, the adjusted mortality risk was lower for adults, both males and females, and all racial and ethnic groups, as well as individuals with and without disabilities, and those with one or more underlying medical conditions during the Omicron period compared to the Delta period (14). In the United Kingdom, a large retrospective cohort study indicated that the risk of COVID-19 mortality was 66% lower for Omicron BA.1 in comparison to Delta (15). Compared to the wild-type virus, the Delta variant has a greater risk of hospitalization, intensive care units admission, and mortality than the Alpha variant, and all SARS-CoV-2 VOCs have a greater risk of disease severity than the wild-type virus (16). During the pre-Omicron period, the proportion of COVID-19-related hospitalizations was relatively stable, whereas it decreased during the Omicron period. Although the predictors of inpatient mortality remained consistent with previous reports, such as older age, male gender, morbid obesity, and multiple comorbidities, it appears that the Omicron variant is a less lethal variant for adults hospitalized with COVID-19, although this effect is weaker in older patients (17). However, it was debated whether Delta was more fatal than Omicron variant for all kinds of populations.

However, the recent COVID-19 Australia Epidemiology Report revealed that patients aged 70–79 and 80–89 years, the mortality rate was 2- and 3-fold, respectively, in the Omicron wave compared with that in the Delta wave (18). Though intensive studies have been conducted to compare the two most infectious variants, Delta and Omicron, data on Alpha variant was relative scarce. In addition, the literature on COVID-19 mortality were partial and specific, either focused on hospitalization rate and mortality rate in different waves of variants; some reported disparities in sex or race but limited to several regions or county level; and some demonstrated the fluctuation of mortality only in early to middle pandemic. The mask wearing policy and vaccination kept rolling by time, while the long-term effect was unclear. A comprehensive interpretation of fluctuations in the case fatality rate (CFR) is of important while absent.

Therefore, the objective of this study was to conduct a longitudinal analysis of CFR trends in relation to COVID-19, while taking into account significant events influencing CFR fluctuations. Furthermore, risk factors for mortality is identified following a COVID-19 diagnosis, and the mortality and hospitalization rates are investigated during the Delta and Omicron waves.

## 2. Methods

### 2.1. Study design

TriNetX is a global federated network that provides access to de-identified electronic medical information from numerous health care organizations, which consist of millions of patients collected from hospital, primary care, and specialist providers. Demographic information, diagnoses, treatments, deaths, prescriptions, and laboratory test results are among the available data. TriNetX aggregates information directly from electronic medical record systems on a continuous basis. Numerous renowned studies employed this database (19–23). Using the TriNetX United States Collaborative Network, we retrieved data from 48 health care organizations in the United States ranged from January 1, 2020 to June 30, 2022. The US network include United States Census Bureau defines four statistical regions, with nine divisions. Region 1: Northeast (Division 1: New England, Division 2: Mid-Atlantic), Region 2: Midwest (Division 3: East North Central, Division 4: West North Central), Region 3: South (Division 5: South Atlantic, Division 6: East South Central, Division 7: West South Central), Region 4: West (Division 8: Mountain, Division 9: Pacific). The data collected by HCOs are representative of their entire patient population and are inclusive of all facilities and treatment settings. Supplementary Table S1 demonstrates Strengthening the Reporting of Observational Studies in Epidemiology (STROBE) Statement.

### 2.2. Study population

The study population consisted of patients diagnosed with COVID-19 in study period. Supplementary Table S1 demonstrates the definition of study group. Patients were included if they met any of the following criteria: (1) International Classification of Diseases, Tenth Revision, Clinical Modification (ICD-10-CM) codes (U07.1, U07.2, U09, U09.9, or J12.82) in medical records; or (2) positive SARS-CoV-2 laboratory test result (codes 94,534-5, 94,309-2, 94,500-6, 94,316-7, or 9,088). There was no restriction on age of participants. The coding was based on the coding guidelines released by Centers for Disease Control and Prevention (CDC). Patients met inclusion criteria will be included regardless of from out-patient visit, in-patient visit, intensive care unit or emergency department. Mortality was estimated following COVID-19 diagnosis until the end of the research. All data were analyzed on the TriNetX platform.

### 2.3. Outcomes

The main focus of this study was to examine the primary outcome of death following infection with SARS-CoV-2. Additionally, we investigated secondary outcomes, which included the occurrence of mortality or hospital inpatient services during the Delta and Omicron waves. Mortality was defined as death within 30 days, while hospital inpatient services were identified through hospitalization records with Current Procedural Terminology (CPT) codes 1,013,659 and 1,013,699 and intensive care unit admission using CPT code 1013729. We calculated the cumulative probability for these outcomes during the follow-up period. Subgroup analysis with time stratification after confirmation of COVID-19 are also conducted.

### 2.4. Statistical analysis

The mortality rate denominator is the monthly number of confirmed COVID-19 case, molecular is the monthly number of deaths in COVID-19. The formula is as below.

Case Fatality Rate%=Monthly number of deaths in COVID−19Monthly number of confirmed COVID−19cases×100

**Figure 1:**
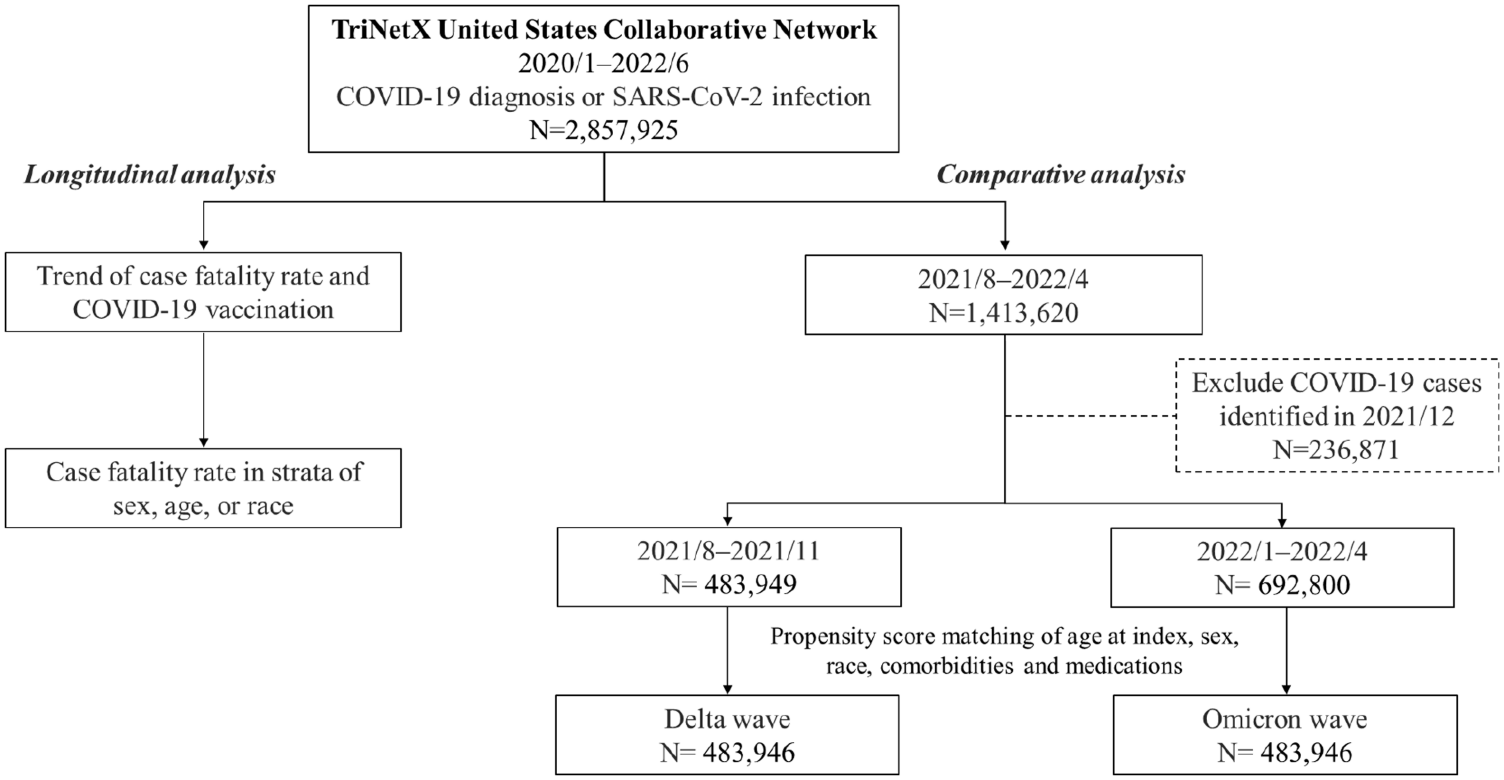
Algorithm of study protocol.

**Figure 2:**
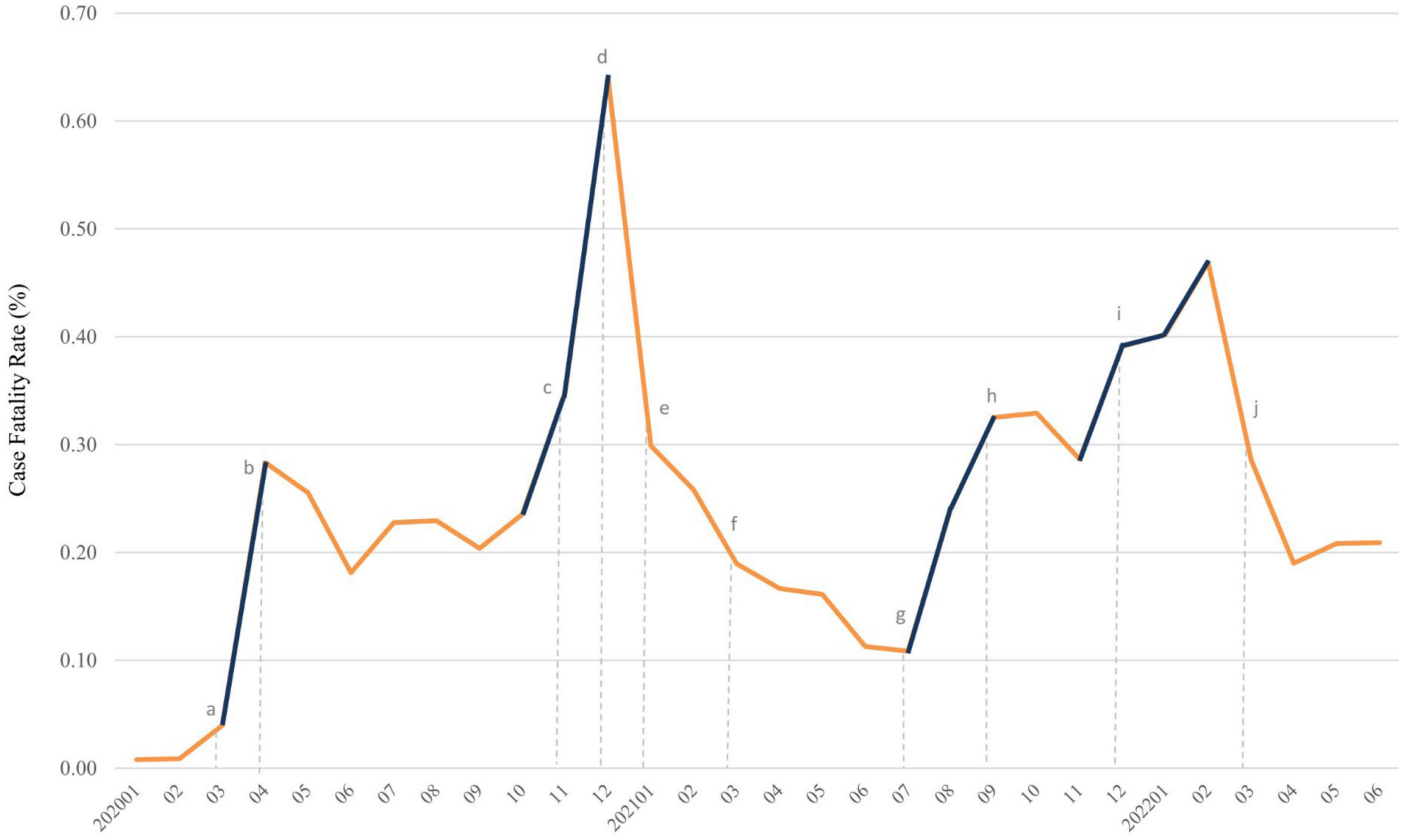
Trends of case fatality rate (%) of COVID-19 from January 2020 to June 2022. Surge of case fatality rate was denoted in deep blue. Dotted line with lowercase letter indicated key event as following: **(A)** outbreak of COVID-19 in the United States, **(B)** mask mandate policy was first implemented, **(C)** appearance of Alpha variant in the United States, **(D)** started COVID-19 vaccination for individuals aged more than 18 years, **(E)** mix-and-match of COVID-19 vaccination was initiated and CDC require mask wearing on public transportation, **(F)** first Delta case was detected in United States, **(G)** Delta variant become dominant in newly diagnosed cases, **(H)** FDA allow booster dose of Pfizer-BioNTech COVID-19 vaccine, **(I)** first Omicron case was detected in United States, and it became dominant variant at end of December, 2021, and **(J)** FDA authorized a second booster dose of Pfizer-BioNTech and Moderna COVID-19 vaccine. CDC, centers for disease control and prevention; COVID-19, coronavirus disease 2019; FDA, food and drug administration.

**Figure 3:**
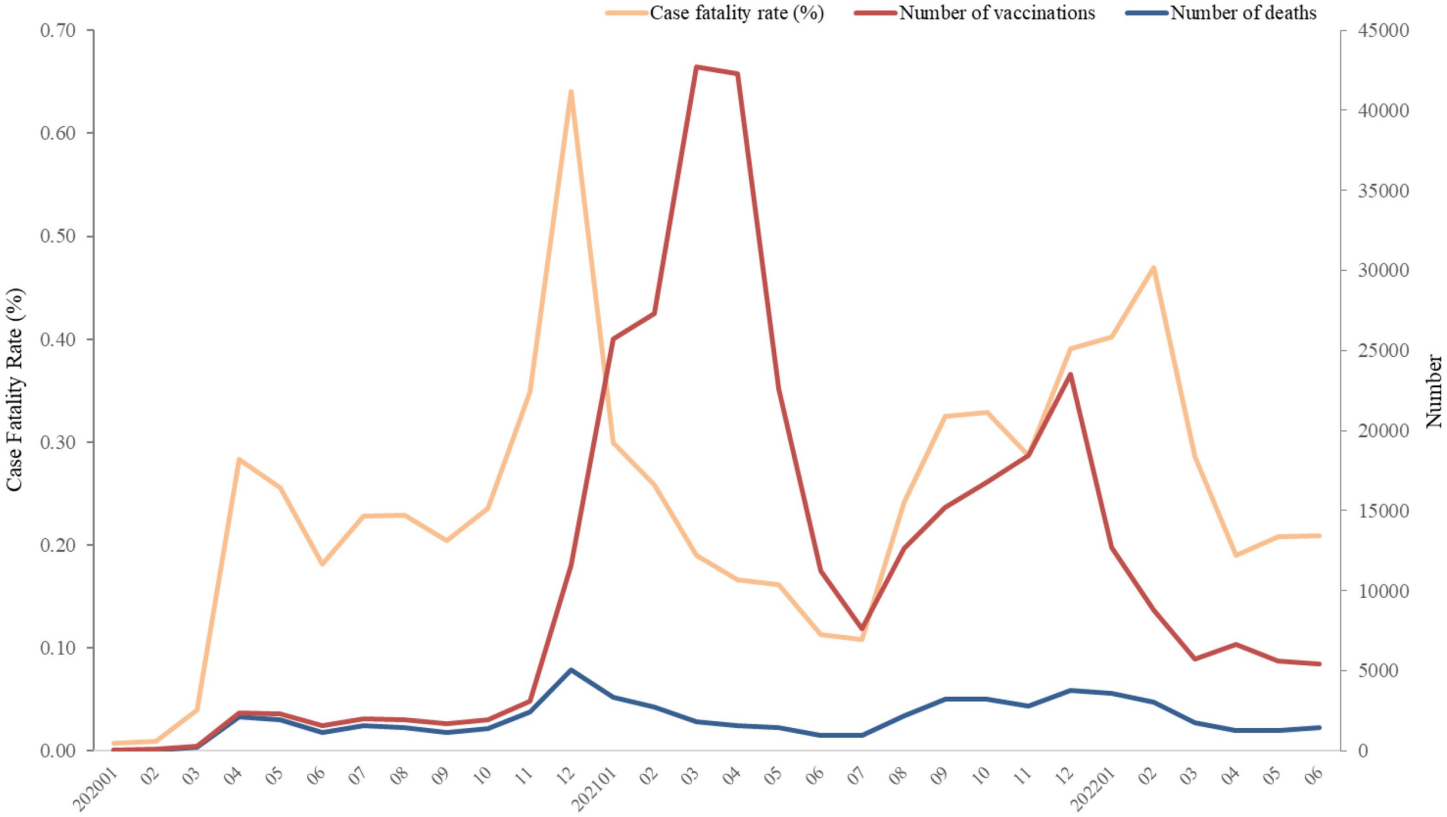
Numbers of deaths and vaccination, and case fatality rate (%) of COVID-19 from January 2020 to June 2022. The number of vaccinations was recorded as a single event, and it was not possible to distinguish between first or second doses due to constraints within the TriNetX platform.

**Figure 4:**
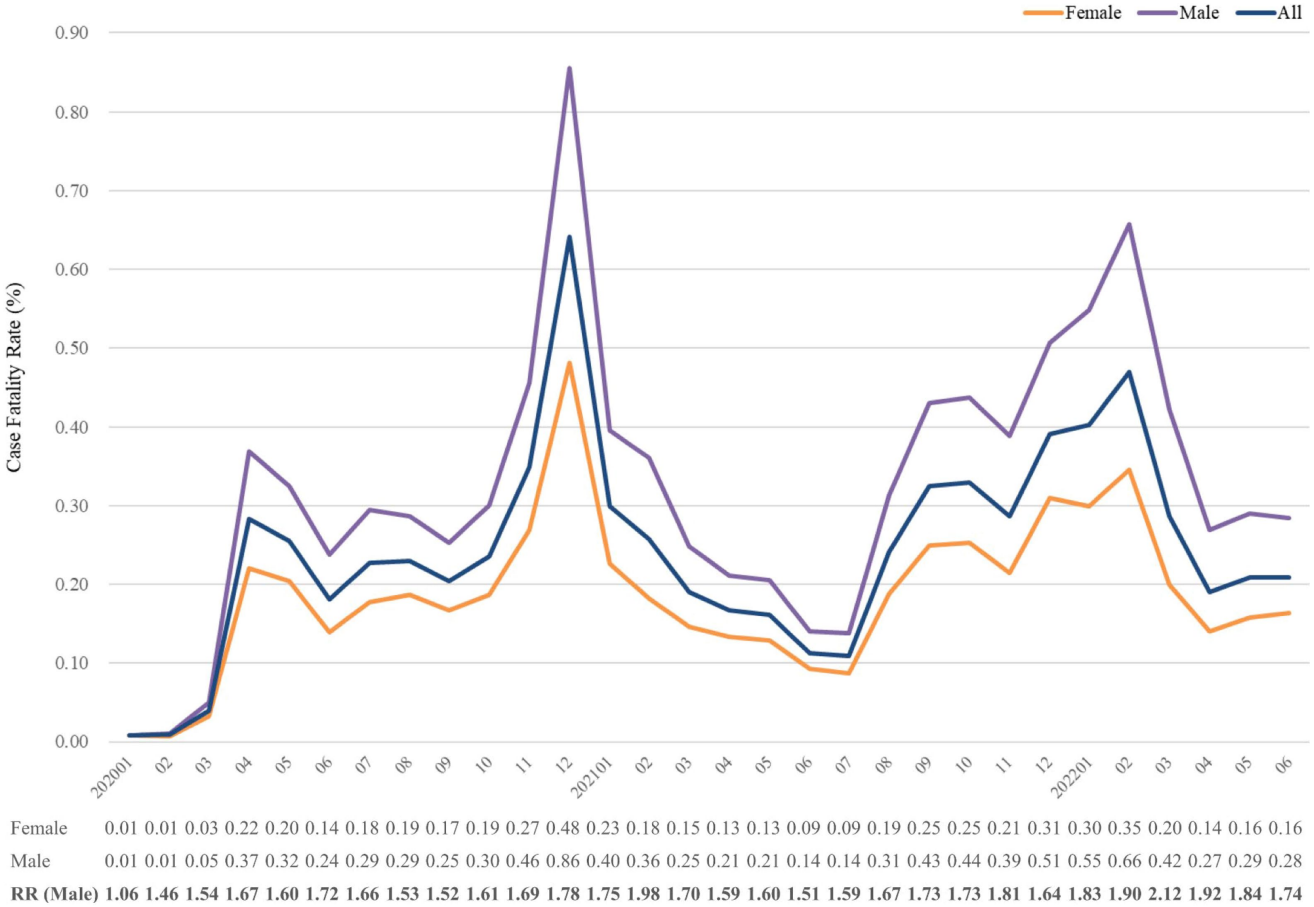
Case fatality rate (%) of COVID-19 from January 2020 to June 2022, stratified by sex. The relative risk of fatality was calculated using the reference group of females. RR, risk ratio.

TriNetX database offered monthly CFR and monthly deaths in COVID-19 without information of monthly number of confirmed COVID-19. After selecting specific period and population with COVID-19 diagnosis, we retrieve CFR on platform. All statistical analyses were performed on TriNetX platform. In Figures 1–4, relative risk was calculated with respect to male participants, individuals aged 19–50 years old, and the Asian population, respectively, serving as references.

Baseline characteristics were analyzed in a descriptive manner to present the number and percentage of individuals with each disease, as well as the mean and standard deviation of age at the index. In order to optimize the relevant covariates between the Delta and Omicron variants, propensity-score matching was employed. The propensity score for each participant was estimated through non-parsimonious multivariable logistic regression, with COVID-19 serving as the dependent variable. We included clinically related covariates as independent variables. The nearest-neighbor algorithm was adopted to construct matched pairs, assuming the standardized mean difference value <0.1 to be a negligible difference between the study and comparison cohorts.

Propensity score matching for the two groups at a 1:1 ratio by greedy nearest neighbor matching for age at index, sex, race, comorbidities (including hypertensive diseases, cerebrovascular diseases, chronic kidney disease, chronic respiratory diseases, diabetes mellitus, dementia, dyslipidemia, ischemic heart diseases, liver diseases, malignancy, overweight or obesity, smoking) and medication (including angiotensin converting enzyme inhibitors or angiotensin II receptor blockers, beta blocking agents, calcium channel blockers, metformin, lipid modifying agents, corticosteroids, non-steroidal anti-inflammatory drugs, and antipsychotics). The propensity score matching procedure employed in the TriNetX platform involves the use of logistic regression analysis to derive propensity scores for each subject based on user-specified covariates. Subsequently, a 1:1 matching is conducted using greedy nearest neighbor algorithms, with a caliper width of 0.1 pooled standard deviations. To mitigate bias resulting from the nearest neighbor algorithms, TriNetX randomizes the order of rows. It is worth noting that this study method has been previously validated (24–26).

Comorbidities that were present within 6 months prior to the index day, which is defined as the date of first COVID-19 diagnosis, will be considered in our analysis. Additionally, we will include commonly used medications that were taken for more than 7 days during the study period. Supplementary Table S2 lists the codes for both comorbidities and medications.

We utilized Cox proportional hazards models to compare the outcomes of interest between the Delta and Omicron variants. Different models of adjustment were used to compare the risks of all-cause mortality and hospitalization during the Delta and Omicron waves. Additionally, we evaluated the risks of death and hospitalization in the Delta wave compared to those in the Omicron wave based on the cumulative duration (<14, 14–30, 31–365 days) following a COVID-19 diagnosis. The results were reported as hazard ratios (HRs) and 95% confidence intervals (CIs) for both Delta and Omicron variants. To calculate the observed risks, we censored the participants until the date of respective outcomes or until the end of follow-up on June 30, 2022, whichever occurred first.

The Kaplan–Meier analysis and log-rank tests were used to estimate the survival probability of primary outcome from day 1 to day 30 post COVID-19 diagnosis during a specific period. TriNetX platform did not report the infected COVID-19 variants, so we determined the interval which is representative of the majority of newly reported cases. Based on variant data by time (27), the Delta and Omicron waves mainly occurred in August–November 2021 and January–April 2022, accounting for at least 99.5 and 98% of the overall number of new cases in the United States, respectively. A subgroup analysis to interval analysis was conducted with age strata. The cumulative probability of mortality resulting from COVID-19 infection in separate waves of variants will also be assessed.

A two-tailed *p*-value of less than 0.05 was regarded as statistically significant. Arrangement of figures and tables were handled on Microsoft Excel version 16.0 (Microsoft, Washington, United States). All statistical analyses were conducted on the TriNetX platform, which utilizes a combination of JAVA^™^, R and Python^™^ programming languages (28).

### 2.5. Acquisition of data and quality control

The TriNetX appliance is either a physical server located in the institution’s data center or a virtual hosted appliance. The TriNetX platform is comprised of a fleet of these appliances connected to a federated network and capable of broadcasting queries to each appliance. Results are collected and aggregated. Once the data have been transmitted to the network, they are mapped to a standard and controlled set of clinical terminologies and undergo a data quality review, including “data cleaning,” which rejects records that do not match TriNetX quality requirements. Compliance with the Health Insurance Portability and Accountability Act (HIPAA) is achieved by de-identifying clinical patient data. TriNetX is certified to the ISO 27001:2013 standard and maintains an Information Security Management System to ensure the protection of the healthcare data it has access to and to meet the requirements of the HIPAA Security Rule. TriNetX employs the standardized methodology for assessing data quality in regard to data quality. Conformance, completeness, and plausibility are recognized as three categories of quality measures in this framework.

### 2.6. Ethical considerations

This study has granted approval by institutional review board of Chung Shan Medical University Hospital with number of CS2-21176. The research consisted exclusively of aggregating de-identified counts and statistical summaries and did not involve the collection or transmission of personally identifiable information; thus, the informed consent was not applicable. To protect privacy and confidentiality of the patients from TriNetX, patient counts are rounded up to the nearest 10 if the count is less than 10.

## 3. Results

### 3.1. Study flowchart

**Figure 5:**
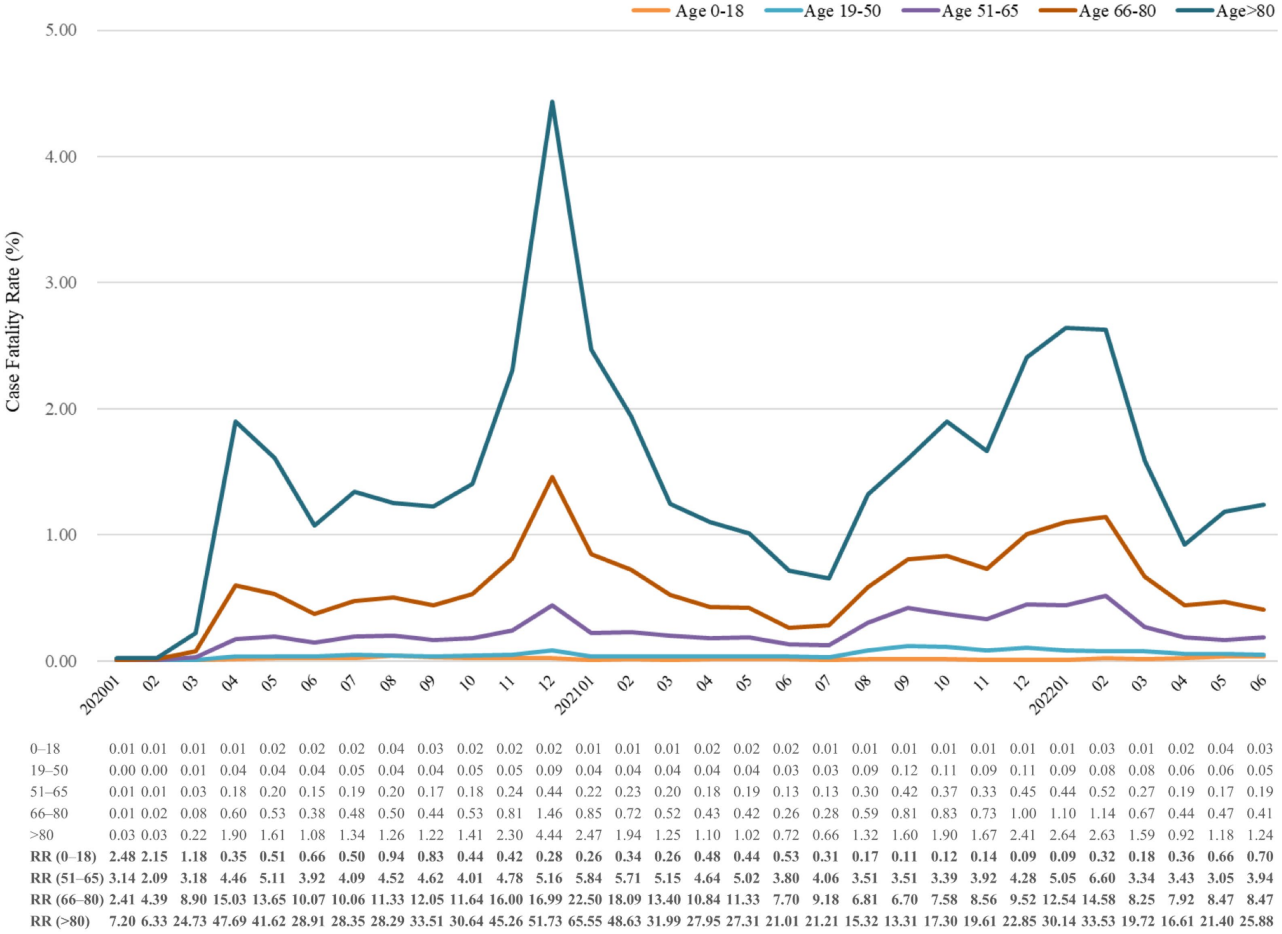
Case fatality rate (%) of COVID-19 from January 2020 to June 2022, stratified by age. The relative risk of fatality was calculated using the reference group of individuals aged 19–50 years old. RR, risk ratio.

Figure 5 presents the algorithm of study protocol. Over the period from January 2020 to June 2022, a total of 2,857,925 individuals were identified as having been diagnosed with COVID-19 or infected with SARS-CoV-2. A longitudinal analysis of the entire cohort population was conducted to examine the secular trend of CFR and COVID-19 vaccination. The time frame chosen for comparative analysis was from August 2021 to April 2022. During this period, a total of 1,413,620 individuals were identified. Among them, 483,949 individuals were identified during the Delta wave, while 692,800 individuals were identified during the Omicron wave. To ensure comparability between the two groups, propensity score matching was conducted at a 1:1 ratio for variables such as age at index, sex, race, comorbidities, and medications. As a result, 483,946 pairs of participants were included in the analysis. The primary focus of this comparative analysis is to examine the mortality and hospitalization rates during the Delta and Omicron waves.

### 3.2. Longitudinal analysis

**Figure 6:**
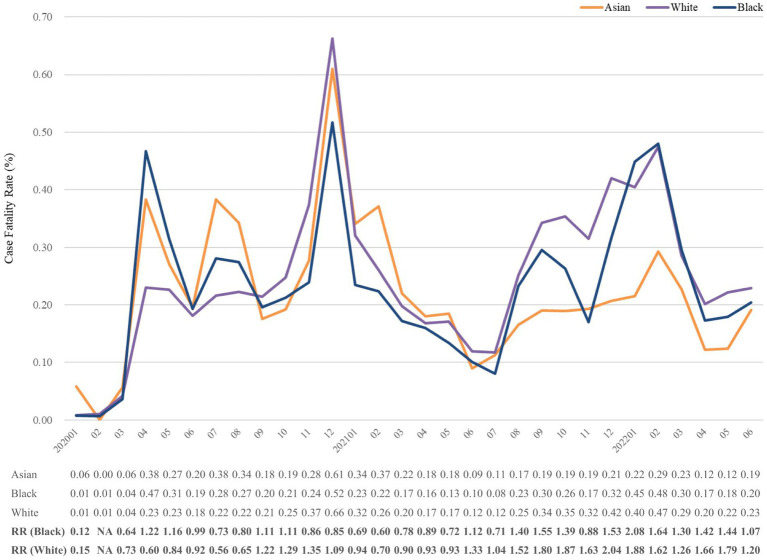
Case fatality rate (%) of COVID-19 from January 2020 to June 2022, stratified by race. The relative risk of fatality was calculated using the reference group of Asian. NA, not available. No record of any death in Asian population in February 2020. Any number lower than 10 would be automatically assigned to numeral 10 in TriNetX; hence, data presentation could be seriously affected. Accordingly, Native Hawaiian, American Indian, and other less common races were not presented.

Figure 6 demonstrates the four sharp surges in CFR, occurring in March–April 2020, October 2020–January 2021, July–September 2021, and November 2021–February 2022. A total of 2,131 deaths were recorded in the first surge, and 5,049 deaths in the second surge (data not shown). The SARS-CoV-2 reached the United States on January 13, 2020 and led to numerous deaths from March to April of 2020. After the implementation of the stay-at-home order on patients diagnosed with COVID-19 on April 3, 2020, the CFR and the number of deaths decreased. Since October 2020, the daily cases of COVID-19 surged, so did CFR. A sharp increase in CFR from November 2020 to January 2021, indicated as the second surge. From January 2021 to July 2021, the CFR sharply declined at a steady level, followed by a noticeable increase. Alpha variant (B.1.1.7) was the first of the highly publicized variants. It rapidly spread and dominated the United States from March to April 2021. However, Alpha diminished with the rise of the more aggressive Delta variant in June 2021. The Delta variant (B.1.617.2) kicked off the third surge from July 2021 to September 2021. The fourth surge, from November 2021 to February 2022, also correlated with the emerging infections caused by the Delta and Omicron variants. The Omicron variant was first detected in the United States on December 1, 2021, and dominated in mid-December 2021.

Figure 1 depicts the secular fluctuations of the number of deaths, vaccinations, and CFR. The highest number of deaths and CFR were recorded in December 2020. Since December 2021, the gap between CFR and number of deaths enlarged. The number of COVID-19 vaccination has dramatically raised since November 2020, whereas the increase was interrupted in February 2021, followed by the same growth rate of vaccination. The number of vaccinations decreased from April to July, 2021.

### 3.3. Secular trend of CFR stratified by sex, age and race

Subgroup analyses with stratification of sex, age and race were conducted. Figure 2 presents the fatality in strata of sex. The mortality trends of both sexes positively correlated with the whole follow-up period. Throughout the study period, the CFR of males was always higher than that of females, and the highest odds of CFR in men over women in March 2022 was 2.12-fold. Of note, the relative risk seemed to be higher in the decreasing stage of CFR, such as July 2020, February 2021, November 2021 and March 2021. It showed that the CFR of males dropped slower than that of females.

Figure 3 shows mortality trends in CFR, which were similar in all aspects of the age group. Age was positively correlated with CFR in study period. Patients aged more than 65 years accounted for a significant number of deaths, but those above 80 years old had the highest CFR for the whole period. In January 2021, the relative risks of CFR were, respectively, 65.55-fold and 22.50-fold higher in people aged above 80 years and 66–80 than in those aged 19–50 years. The CFR of age 0–18 years remained lower than 1 except for the first three consecutive months of the study. Intriguingly, the relative risk dramatically increased in older age groups when CFR went up, which may indicate that older people were more vulnerable to the pandemic.

Supplementary Figure S1 demonstrates the CFR in strata of sex and age. Male sex and age seemed to be strong and independent risk factors of mortality in COVID-19. Secular trend in CFR with stratification of sex and age also indicated overall higher risk in male sex and older age. Whereas for age group 0–18, the number of deaths were very low in stratification, hence the disparity of CFR between both sexes were not prominent.

Figure 4 indicates the CFR trend in strata of race. The CFRs of Asian, White, and Black groups positively correlated with each other until July 2021. Subsequently, the CFR of Asians remained lower than the others. The highest relative risks were observed in the fourth surge, 2.04 and 2.08 of White and Black, respectively (See Table 1).

**Table 1:** Baseline characteristics of included participants.

	Before PSM			After PSM		
	Delta *N* = 483,949	Omicron *N* = 692,800	SMD	Delta *N* = 483,946	Omicron *N* = 483,946	SMD
Number	483,949	692,800		483,946	483,946	
Age at index (Mean ± SD)	40.9 ± 22.5	41.0 ± 22.8	0.002	40.9 ± 22.5	40.9 ± 22.5	0.003
Sex						
Female	268,668 (55.5%)	403,292 (58.2%)	0.054	268,666 (55.5%)	268,828 (55.5%)	0.001
Male	215,049 (44.4%)	289,219 (41.7%)	0.054	215,048 (44.4%)	214,957 (44.4%)	<0.001
Race						
White	327,014 (67.6%)	442,438 (63.9%)	0.078	327,014 (67.6%)	324,843 (67.1%)	0.01
Black or African American	75,465 (15.6%)	113,851 (16.4%)	0.023	75,465 (15.6%)	76,243 (15.8%)	0.004
Asian	7,468 (1.5%)	17,394 (2.5%)	0.069	7,465 (1.5%)	7,956 (1.6%)	0.008
American Indian or Alaska Native	2017 (0.4%)	2,758 (0.4%)	0.003	2017 (0.4%)	1895 (0.4%)	0.004
Native Hawaiian or other Pacific Islander	737 (0.2%)	1,218 (0.2%)	0.006	737 (0.2%)	753 (0.2%)	0.001
Unknown	71,248 (14.7%)	115,141 (16.6%)	0.052	71,248 (14.7%)	72,256 (14.9%)	0.006
Comorbidities						
Hypertensive diseases	68,190 (14.1%)	108,096 (15.6%)	0.043	68,187 (14.1%)	64,761 (13.4%)	0.021
Cerebrovascular diseases	7,390 (1.5%)	12,570 (1.8%)	0.022	7,388 (1.5%)	6,959 (1.4%)	0.007
Chronic kidney disease	14,231 (2.9%)	24,650 (3.6%)	0.035	14,229 (2.9%)	13,279 (2.7%)	0.012
Chronic respiratory diseases	30,604 (6.3%)	51,818 (7.5%)	0.046	30,604 (6.3%)	29,733 (6.1%)	0.007
Diabetes mellitus	34,550 (7.1%)	53,922 (7.8%)	0.025	34,548 (7.1%)	32,149 (6.6%)	0.02
Dementia	1788 (0.4%)	3,382 (0.5%)	0.018	1788 (0.4%)	1714 (0.4%)	0.003
Dyslipidemia	52,806 (10.9%)	83,004 (12%)	0.034	52,803 (10.9%)	50,056 (10.3%)	0.018
Ischemic heart diseases	17,354 (3.6%)	27,751 (4%)	0.022	17,353 (3.6%)	16,064 (3.3%)	0.015
Liver diseases	8,442 (1.7%)	14,531 (2.1%)	0.026	8,441 (1.7%)	7,952 (1.6%)	0.008
Malignancy	28,756 (5.9%)	48,580 (7%)	0.043	28,753 (5.9%)	28,187 (5.8%)	0.005
Overweight or obesity	30,265 (6.3%)	48,106 (6.9%)	0.028	30,264 (6.3%)	28,665 (5.9%)	0.014
Smoking	3,424 (0.7%)	5,562 (0.8%)	0.011	3,424 (0.7%)	3,190 (0.7%)	0.006
Medications						
ACEis/ARBs	31,563 (6.5%)	50,429 (7.3%)	0.03	31,561 (6.5%)	29,613 (6.1%)	0.017
Beta blocking agents	30,282 (6.3%)	50,203 (7.2%)	0.039	30,280 (6.3%)	28,343 (5.9%)	0.017
Calcium channel blockers	20,282 (4.2%)	35,224 (5.1%)	0.042	20,279 (4.2%)	19,270 (4%)	0.011
Metformin	12,422 (2.6%)	19,104 (2.8%)	0.012	12,421 (2.6%)	11,362 (2.3%)	0.014
Lipid modifying agents	34,486 (7.1%)	56,835 (8.2%)	0.041	34,483 (7.1%)	32,326 (6.7%)	0.018
Corticosteroids	42,494 (8.8%)	72,797 (10.5%)	0.059	42,492 (8.8%)	42,077 (8.7%)	0.003
Non-steroidal anti-inflammatory drugs	51,500 (10.6%)	80,314 (11.6%)	0.03	51,498 (10.6%)	50,518 (10.4%)	0.007
Antipsychotics	15,524 (3.2%)	27,263 (3.9%)	0.039	15,521 (3.2%)	15,193 (3.1%)	0.004

ACEis, angiotensin converting enzyme inhibitors; ARBs, angiotensin II receptor blockers; PSM, propensity score matching; SMD, standardized mean difference.

### 3.4. Comparative analysis of Delta and Omicron

We evaluated all-cause mortality and hospitalization rates during the Delta and Omicron waves. Table 2 presents hazards of all-cause mortality and hospitalization during Delta wave over Omicron wave in various models. The 14 days cumulative probability of all-cause mortality was 1.20 and 0.69% during the Delta and Omicron waves, respectively. At 1 year, the probabilities increased to 3.82 and 2.80% for Delta and Omicron, respectively. The crude HR for Delta was 1.336 times higher than for Omicron. Regarding hospitalization, the 14 days cumulative probability was 10.90 and 7.36% for Delta and Omicron, respectively. At 1 year, these probabilities increased to 21.94 and 20.86% for Delta and Omicron, respectively. The crude HR for Delta was 1.099 times higher than for Omicron. After adjusting for age at index, sex, race, and comorbidities, the hazard ratios for all-cause mortality and hospitalization were 1.453 and 1.146 times higher for the Delta variant compared to the Omicron variant, respectively.

**Table 2:** Risks of all-cause mortality and hospitalization during Delta and Omicron wave.

	Cumulative probability (%)			Crude HR* (95% CI)	Model 1	Model 2	Model 3
	14 days	30 days	365 days				
All-cause mortality							
Delta	1.20%	2.13%	3.82%	1.336 (1.305–1.368)	1.362 (1.327–1.398)	1.453 (1.415–1.493)	1.453 (1.414–1.492)
Omicron	0.69%	1.12%	2.80%	Reference	Reference	Reference	Reference
Hospitalization							
Delta	10.90%	12.12%	21.94%	1.099 (1.089–1.109)	1.101 (1.091–1.112)	1.14 (1.128–1.151)	1.146 (1.134–1.157)
Omicron	7.36%	8.71%	20.86%	Reference	Reference	Reference	Reference

*Hazard ratio of all-cause mortality or hospitalization among Delta wave compared with Omicron wave after prosperity score matching (PSM). HR, hazard ratio; 95% CI, 95% confidence interval. Model 1: PSM by age at index, sex, and race. Model 2: PSM by age at index, sex, race and comorbidities. Model 3: PSM by age at index, sex, race and comorbidities and medication.

Table 3 shows time stratified analyses, which revealed adjusted hazard ratios (Model 3) of death and hospitalization during the Delta wave were 1.453 and 1.146 times higher than Omicron wave, respectively, in a year after COVID-19 diagnosis. Different models revealed consistent findings. The risk of death was notably elevated at 14–30 days after COVID-19 diagnosis, with a risk ratio of 2.194 (95% CI 2.071–2.323). On the other hand, the risk of hospitalization was notably elevated in the first 14 days after COVID-19 diagnosis (adjusted HR 1.485, 95% CI 1.464–1.505).

**Table 3:** Risks of all-cause mortality and hospitalization in strata of period after COVID-19 diagnosis.

	Crude HR* (95% CI)	Model 1	Model 2	Model 3
1–365 days				
All-cause mortality	1.336 (1.305–1.368)	1.362 (1.327–1.398)	1.453 (1.415–1.493)	1.453 (1.414–1.492)
Hospitalization	1.099 (1.089–1.109)	1.101 (1.091–1.112)	1.14 (1.128–1.151)	1.146 (1.134–1.157)
<14 days				
All-cause mortality	1.656 (1.588–1.727)	1.707 (1.628–1.79)	1.742 (1.661–1.827)	1.731 (1.651–1.816)
Hospitalization	1.439 (1.421–1.457)	1.434 (1.414–1.454)	1.486 (1.465–1.507)	1.485 (1.464–1.505)
14–30 days				
All-cause mortality	2.067 (1.966–2.172)	2.108 (1.993–2.231)	2.194 (2.072–2.324)	2.194 (2.071–2.323)
Hospitalization	1.233 (1.208–1.258)	1.216 (1.189–1.242)	1.294 (1.265–1.323)	1.306 (1.277–1.335)
31–365 days				
All-cause mortality	0.952 (0.919–0.986)	0.973 (0.937–1.01)	1.07 (1.029–1.112)	1.073 (1.032–1.116)
Hospitalization	0.848 (0.838–0.858)	0.851 (0.84–0.862)	0.887 (0.876–0.898)	0.896 (0.885–0.908)

*Hazard ratio of all-cause mortality or hospitalization among Delta wave compared with Omicron wave after prosperity score matching (PSM). HR, hazard ratio; 95% CI, 95% confidence interval. Model 1: PSM by age at index, sex, and race. Model 2: PSM by age at index, sex, race and comorbidities. Model 3: PSM by age at index, sex, race and comorbidities and medication.

Supplementary Table S3 shows five VOCs have been identified in the United States. Supplementary Table S4 summarizes the impact of the variants on transmissibility, hospitalization, mortality, and efficacy of the vaccines. To investigate mortality in Delta, and Omicron waves, we analyzed separate periods: August–November 2021 and January–April 2022. During these periods, the abovementioned variants dominated and accounted for majority of the newly diagnosed cases.

### 3.5. Kaplan–Meier analysis

**Figure 7:**
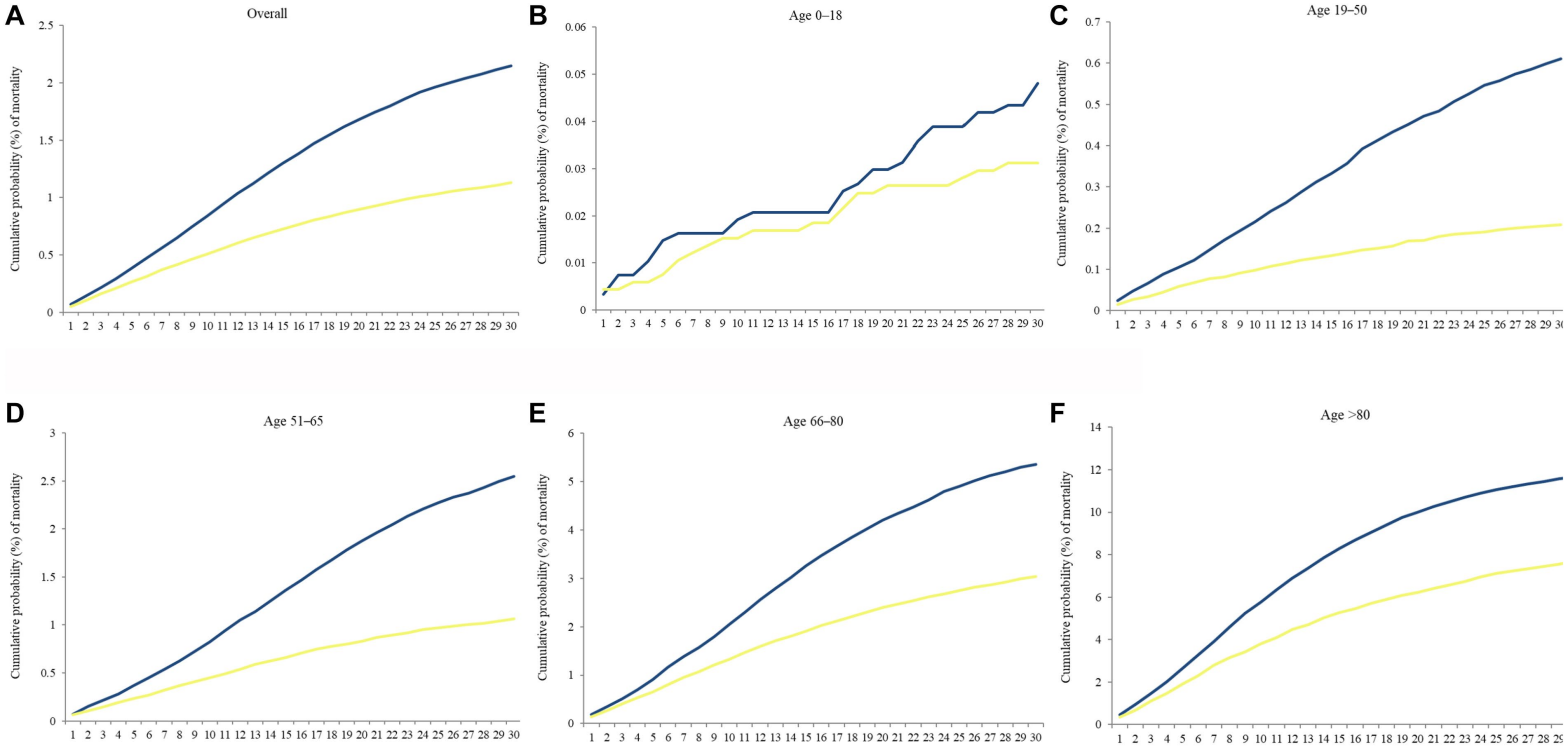
Kaplan–Meier curves of the 30 days cumulative probability of mortality in separate periods, August–November 2021 (Delta wave) and January–April 2022 (Omicron wave), stratified by age. **(A)** Overall, **(B)** Age 0–18, **(C)** Age 19–50, **(D)** Age 51–65, **(E)** Age 66–80, and **(F)** Age > 80. Blue line denoted Delta wave, and yellow line denoted Omicron wave.

Figure 7 reveals Kaplan–Meier curves indicating 30 days cumulative probability of mortality stratified by age. The Delta to Omicron variants were subjected to a log-rank test. The curves for those aged 0–18 years were jagged due to the limited number of events. Table 4 displays the cumulative probability of mortality for different variants, divided by age groups. The cumulative probability of mortality was approximately 2-fold on day 30 in Delta than in Omicron cases (log-rank *p* < 0.001). The overall mortality risks of Delta and Omicron variants were, respectively, 2.15 and 1.13%. The risk of deaths increased with ages in all waves of variants. The relative risk of Delta to Omicron was 1.671 (95% Cl 1.615–1.729, log-rank *p* < 0.001). Delta variant was most fatal for individuals aged 19–50 years compared with Omicron variant (relative risk 2.885, 95% Cl 2.544–3.271). Supplementary Figure S2 shows the CFR of each age group across the study period. The CFR of patients aged above 80 years was as high as 17.33%, followed by those aged 66–80 years as 6.97%.

**Table 4:** Relative risk of 30 days cumulative probability of mortality during August–November 2021 (Delta wave) and January–April 2022 (Omicron wave), stratified by age.

Variables	Cumulative probability of mortality			Log-rank *p*
	Risk of death	cHR (95% CI)	aHR (95% CI)	
Overall				
Delta	2.15%	1.788 (1.731–1.847)	1.896 (1.827–1.968)	<0.001
Omicron	1.13%	Reference	Reference	
Age 0–18				
Delta	0.05%	1.09 (0.689–1.724)	1.499 (0.867–2.591)	0.209
Omicron	0.03%	Reference	Reference	
Age 19–50				
Delta	0.61%	2.89 (2.592–3.221)	2.885 (2.544–3.271)	<0.001
Omicron	0.21%	Reference	Reference	
Age 51–65				
Delta	2.54%	2.231 (2.083–2.39)	2.38 (2.199–2.577)	<0.001
Omicron	1.06%	Reference	Reference	
Age 66–80				
Delta	5.36%	1.722 (1.634–1.815)	1.775 (1.672–1.883)	0.016
Omicron	3.04%	Reference	Reference	
Age > 80				
Delta	11.66%	1.481 (1.394–1.573)	1.543 (1.44–1.652)	<0.001
Omicron	7.71%	Reference	Reference	

aHR, adjusted hazrad ratio; cHR, crude hazard ratio; 95% Cl, 95% confidence interval.

## 4. Discussion

This is macroscopic and longitudinal review on mortality during the COVID-19 pandemic in the United States. It enhances the findings of previous studies by providing considerably more detailed perspectives, especially the cumulative probability of mortality and hospitalization in Delta and Omicron waves. The fluctuating mortality trends in the present study were grossly consistent with the data from the COVID Data Tracker based on the whole population.

### 4.1. Pivotal factors influence case fatality rate

Nonpharmaceutical interventions (NPIs) are the most effective public health interventions against COVID-19 (29, 30). NPIs include maintaining hand hygiene, social distancing, wearing face masks, and quarantine. Through NPI implementation, the high mortality trend was reversed by late April 2020, and the CFR was maintained below 10%. High adherence to mask wearing may successfully hinder the increase in the number of COVID-19 cases (31). Wearing face masks in public is the most effective means against COVID-19 before the development of vaccines (32). COVID-19 incidence decreased in 24 counties with mask mandates in public settings while it continued to rise in 81 counties without mask requirements (33).

As multiple countries have implemented age restrictions for the ChAdOx1 n-CoV-19 vaccine (AstraZeneca) since January 2021 due to the emergence of vaccine-induced thrombocytopenia and thrombosis, and the immunization of young people with mRNA vaccines has been halted due to concerns of myocarditis (34, 35). These safety concerns have led to vaccination hesitancy, thereby hindering the growth rate of vaccination in mid-February 2021 (36). During this time period, both the vaccination growth rate and the CFR decline rate decreased.

### 4.2. Mortality by sex

The mortality rates of COVID-19 in the United States were higher in males than in females (37, 38). Similar to our findings, disparity of sex in COVID-19 outcomes have also been documented (39). Throughout the study period, CFR of males was always higher than females. The relative risk of male over female was elevated on decreased portion of CFR rather than the uprising portion, such as 1.72, 1.98, 1.81, and 2.12 corresponding to June 2020, February 2021, November 2021, and March 2022. Although rate of full immunization increased by time, the disparity of CFR between male and female did not diminish.

Sex-based disparities have been widely reported. Females seem to develop stronger innate and adaptive immune responses toward infection and vaccination than males (40). Epidemiological data strongly indicate that women having lower infection rate and (41) hospitalization rates, coupled with better prognosis and lesser mortality (42, 43). This disparity in disease burden may be accounted for by several mechanisms, including sex hormones, genetic factors, differences in immune responses, discrepancy in social behavior, and concurrent comorbidities (44–46).

### 4.3. Mortality by age

In the present study, the CFR of person aged below 18 years old was extremely low compared with other age groups. According to CDC statistics, the crude risks of hospitalization, intensive care unit admission, and mortality remained extremely low for adolescents aged 12–17 (47). BNT162b2 vaccination also reduced the two-third of the risk in Omicron-associated hospitalization among children aged 5–11 years (48). Despite an increase in the vaccination rate among children, the CFR of children has risen dramatically. The cause for this increase was unclear. We did not know if infected children or adolescents contributed to this phenomenon because the 0–18 age group was not further subdivided. The multisystem inflammatory syndrome in children (MIS-C) may be one of the underlying causes (49). The most recent CDC data on pediatric epidemiology found that 60–70% of MIS-C patients were admitted to critical care, with a 1–2% mortality rate (50).

Despite mortality surge in people aged over 50 years, the notification rates and CFR had decreased since July 2021, possibly because of the initiation of vaccination policy and less comorbidity compared with older age. People who aged above 80 years had the highest CFR after COVID-19 diagnosis. In addition, age is always positively correlated to CFR across 4 surges, indicating that age is an independent risk factor of COVID-19-related mortality. Intriguingly, the mortality did not decrease sharply at every surge as the age groups of 66–80 and above 80 years.

### 4.4. Mortality by race

In the United States, marked racial and ethnic inequities in COVID-19 CFR were detected, and this finding remains up to this day (51). Black people bear a disproportionate burden of COVID-19-related consequences in geographic regions where statistics by race/ethnicity have been recorded. The pandemic revealed health disparities and presented a chance to address the underlying causes of these disparities (52). In nursing homes, White has a disproportionally high CFR than Black and Asian (53, 54). Nonetheless, 33% of hospitalized patients were Black, despite they make up only 13% of the United States population (55). A highest age-adjusted mortality rates of Black people was detected, especially Black men (39, 56). In the current analysis, there was no significant difference between White’s and Black’s CFR, with the exception of November 2021. The mortality risk ratio between White and Black was approximately 2:1.

The most common explanations for a disproportionate burden entail certain issues. First, the prevalence of underlying comorbidities is disproportionately high among populations of ethnic minorities. Second, vaccination against COVID-19 was implemented unequally. Vaccine uptake was significantly lower among Black than White (57). On April 21, 2021, the percentage of the total population that had received at least 1 dose of COVID-19 was 33, 32, and 20% in Asian, White, and Black populations but became 65, 48, and 36% after 3 months, respectively (58). The CFRs of Asian and White were similar from the COVID-19 outbreak until and after July 2021, coinciding with the growing differences in COVID-19 vaccination in both races. Third, the disparity of culture between Western and Eastern may affect the perspectives on mask wearing, which may also affect the mortality on some scale (59).

### 4.5. Delta and Omicron waves

We included the Delta and Omicron variants into our comparative analysis due to their significant infectivity and transmissibility. These variants have been a public health concern for an extended period of time. There are a few reasons for Delta spreading rapidly despite high vaccination coverage. First, Delta variant is considerably more transmissible (1.97-fold transmissibility with wild-type SARS-CoV-2) than previous strains (60). Second, immunity against COVID-19 wears off months after vaccination. Third, recent higher rates of infection may partially be explained that more people have undergone COVID-19 test. Fourth, the mutation of molecules on the surface of the virus may significantly decrease 40% of the vaccine’s efficacy and safety after completing a primary series of vaccinations (61). Finally, the rapid Delta spread largely reflected the uneven vaccination coverage and policy of NPIs (62, 63).

Before mid-November, 2022, approximately 1% of the reported COVID-19 cases were reinfected, but now, the rate has increased to around 10%. Since the first detection of the Omicron variant, the reinfection rate has been growing rapidly, whereas this trend was not presented before. Previous infection is 90% effective against infection with the Alpha, Beta, or Delta variants, but only 56% effective against the Omicron variant (64). This result may explain why even a population with a high full-vaccination rate still experienced a higher CFR during the fourth surge than the previous Delta outbreak. Breakthrough infections may still occur in fully vaccinated people with serious outcomes (65, 66). Omicron cases showed a 50–70% reduced risk of hospitalization, a 44% lower chance of any hospitalization, and a 69% lower risk of death compared to Delta cases (67, 68). However, the ratio of per-week Omicron to Delta incidence rate for high mortality was 3.34-fold (69). Even in a highly vaccinated population, highly transmissible SARS-CoV-2 variants may still lead to substantial mortality.

### 4.6. Mortality in Delta and Omicron waves

In England, Beaney et al. (70) reported a 28 days increase in CFR across all age groups, peaking in Delta waves in different age groups before declining. Sigal et al. (71) further proved that the CFR of Omicron was approximately 2.0-fold lower than that of Delta. Though the incidence rate of COVID-19 during the Omicron wave was 6–8 times higher than during the Delta wave (72), the CFR of Omicron was relatively lower. A previous study showed that severity based on the World Health Organization Clinical Progression Scale was 0.61-fold lower for Omicron than for Delta, which was consistent with our findings (73). We identified the 30 days cumulative probability of mortality was 0.53-fold lower in Omicron wave than that in Delta wave.

In Australia, Omicron waves obtained higher mortality rates than Delta waves in all age groups (18). For patients aged 70–79 and 80–89 years, the mortality risk was 2- and 3-fold, respectively, in the Omicron wave compared with that in the Delta wave (18). By the end of 2021, the percentage of completion of primary, secondary, and booster doses in Australia was 80, 77, and 9.3%, respectively, compared with 74, 63, and 23% in the United States (74). No significant difference in percentage of immunization was observed. New COVID-19 cases did not remarkably increase until the end of December 2021 in Australia, but a tremendous number of cases had emerged since March 2020 in the United States. It may be the major reason for the mismatching mortality because of gain of immunity against COVID-19 after recovery of SARS-CoV-2 infection, in conjunction with vaccination can had added protection.

### 4.7. Hospitalization in Delta and Omicron waves

According to several studies, hospitalization rates during the Omicron wave were higher than during the Delta wave, but patients hospitalized with Omicron experienced less severe outcomes compared to those infected with Delta (67, 75–77). Specifically, Omicron-related hospitalizations were less likely to require high flow oxygen, positive pressure ventilatory support, or more critical care, and to have a hospital stay lasting more than 3 days (67, 77, 78). Furthermore, fewer hospitalized patients with Omicron developed severe hypoxemia than those with Delta (adjusted HR 0.55, 95% Cl 0.38–0.78) (75). However, our finding indicated that cumulative probability and risk of hospitalization were both higher in Delta wave than those in Omicron wave. An England cohort study supported our findings that confirmed Omicron cases had a lower risk of hospital admission and any hospital attendance compared to Delta cases (67). Greene et al. (79) founded the risk of hospitalization was lower for New York patients who tested positive for Omicron than for those who tested positive for Delta.

### 4.8. Strengths and limitations

This study is the first to comprehensively analyze COVID-19 mortality with longitudinal presentation since the COVID-19 outbreak, with a subgroup analysis of sex, age, and race. The mortality and hospitalization following COVID-19 diagnosis was also identified in separate waves of predominant variants. Moreover, we systemically reviewed the trends of mortality through an intensive review of the literature and current events. Some limitations need to be addressed. First, TriNetX uses electronic medical record data rather than population-based data. Thus, the infection rate and mortality rate were difficult to define; only CFR can be retrieved on the TriNetX database. Second, epidemiological datasets shared the same caveat that the most recent data are always incomplete because reports of the data of people who died today are yet to be finished for many days, weeks, or even months. We found that number of deaths from March 2022 to June 2022 was different at separate access time to the database; one for August 2022, another was April 2023. The latter should be more corrected. Third, CFR is the main parameter assessed in the present study. Of note, when attempting to calculate the CFR, the probability of a substantial number of unreported cases during the earliest phases of the outbreak may exist. Not only the most recent data, but the earliest data in the pandemic may be biased. Fourth, our study population was limited to specific health care organizations; however, the findings may be applicable to the entire US population. The current findings support previous data and the systemic review of the secular trend of CFR caused by COVID-19 and its numerous variants. Fifth, incidence rate could not be obtained on TriNetX platform due to the unavailability of person-year or person-month data. Consequently, we employed the method of calculating cumulative probability to approximate the risks associated with death or hospitalization. Sixth, due to the inability to incorporate vaccinated status as a variable in propensity score matching on platform, it was not feasible to assess the impact of COVID-19 vaccination. Seventh, the severity of COVID-19 could not be well identified on the platform and hinder further analyses. Lastly, during the early Omicron wave, a small number of deaths may have been caused by infections with the Delta variant that happened several weeks earlier, but we cannot confirm it because of the absence of coronavirus sequencing data.

## 5. Conclusion

Over the course of 2 1/2 years, our analysis of real-world data has revealed significant disparities in COVID-19 CFRs in the United States across sex, age and race. We found that advanced age and male gender were both strong predictors of COVID-19 mortality. With regards to SARS-CoV-2 VOCs, concerns center around viral transmissibility, disease severity, and the impact on vaccine efficacy. Preventing COVID-19 deaths, particularly among individuals who are at higher risk, continues to be a crucial public health objective that requires prioritizing vaccination, early treatment, and appropriate non-pharmaceutical interventions.

Our analysis found that the Delta variant was associated with higher hospitalization and mortality rates compared to the Omicron variant, indicating a greater likelihood of severe disease and poorer outcomes. Among the most vulnerable populations during the COVID-19 pandemic were individuals between the ages of 19 and 50, with a significantly increased risk of hospitalization within 14 days and death within 14–30 days following SARS-CoV-2 infection.

## Data availability statement

The data analyzed in this study is subject to the following licenses/restrictions: once you log in TriNetX live dataset, you can access the raw data utilized in this study. Requests to access these datasets should be directed to https://live.trinetx.com/.

## Ethics statement

This study was approved by institutional review board of Chung Shan Medical University Hospital with number of CS2-21176. This retrospective study is exempt from informed consent. The data reviewed is a secondary analysis of existing data, does not involve intervention or interaction with human subjects, and is de-identified per the de-identification standard defined in Section §164.514(a) of the HIPAA Privacy Rule. The study consisted exclusively of aggregating de-identified counts and statistical summaries and did not involve the collection or transmission of personally identifiable information. To protect privacy and confidentiality of the patients from TriNetX, patient counts are rounded up to the nearest 10 if the count is less than 10.

## Author contributions

J-XL and P-LL had full access to all of the data in the study and take responsibility for the integrity of the data and the accuracy of the data analysis. J-XL: concept and design, interpretation of data, and drafting of the manuscript. P-LL: acquisition and analysis of data and statistical analysis. J-XL, C-JY, P-LL, JC-CW, S-BH, and C-JY: critical revision of the manuscript for important intellectual content. C-JY: supervision. All authors contributed to the article and approved the submitted version.

## Funding

This research received funding support by the Chung Shan Medical University Hospital (CSH-2022-A-015).

## Conflict of interest

The authors declare that the research was conducted in the absence of any commercial or financial relationships that could be construed as a potential conflict of interest.

## Publisher’s note

All claims expressed in this article are solely those of the authors and do not necessarily represent those of their affiliated organizations, or those of the publisher, the editors and the reviewers. Any product that may be evaluated in this article, or claim that may be made by its manufacturer, is not guaranteed or endorsed by the publisher.

## Abbreviations

CDC: Centers for disease control and prevention
CFR: Case fatality rate
COVID-19: Coronavirus disease 2019
EUA: Emergency use authorization
FDA: Food and drug administration
HIPAA: Health Insurance Portability and Accountability Act
MIS-C: Multisystem inflammatory syndrome in children
SARS-CoV-2: Severe acute respiratory syndrome coronavirus 2
VOC: Variants of concern
