# Supplementary material for: A chronological review of COVID-19 case fatality rate and its secular trend and investigation of all-cause mortality and hospitalization during the Delta and Omicron waves in the United States: a retrospective cohort study
Source: Front Public Health. 2023 Sep 15;11:1143650. doi: 10.3389/fpubh.2023.1143650 (PMC10548482; doi:10.3389/fpubh.2023.1143650)
Supplement: Supplementary file 1 [file Data_Sheet_1.PDF]

Supplementary Materials

Study title:

A Chronological Review of COVID-19 Case Fatality Rate and its Secular Trend and Investigation of All-cause Mortality and Hospitalization during the Delta and Omicron Waves in the United States: A Retrospective Cohort Study.

Supplementary Figures

Supplementary Figure 1. Case fatality rate (%) of COVID-19 from January 2020 to June 2022, stratified by sex and age.

Supplementary Figure 2. Bubble plot of overall case fatality rate (%) versus age groups 0–18, 19–50, 51–65, 66–80 and more than 80.

Supplementary Tables

Supplementary Table 1. STROBE statement—checklist of items that should be included in reports of observational studies.

Supplementary Table 2. Search strategy and definition of study group.

Supplementary Table 3. Variants of concern in the United States.

Supplementary Table 4. Impact of COVID-19 variants on transmissibility, hospital admission, mortality, and vaccination efficacy profile against variants

.

Supplementary Figure 1. Case fatality rate (%) of COVID-19 from January 2020 to June 2022, stratified by sex and age. (A) Age 0–18, (B) Age 19–50, (C) Age 51–65, (D) Age 66–80, (E) Age >80.

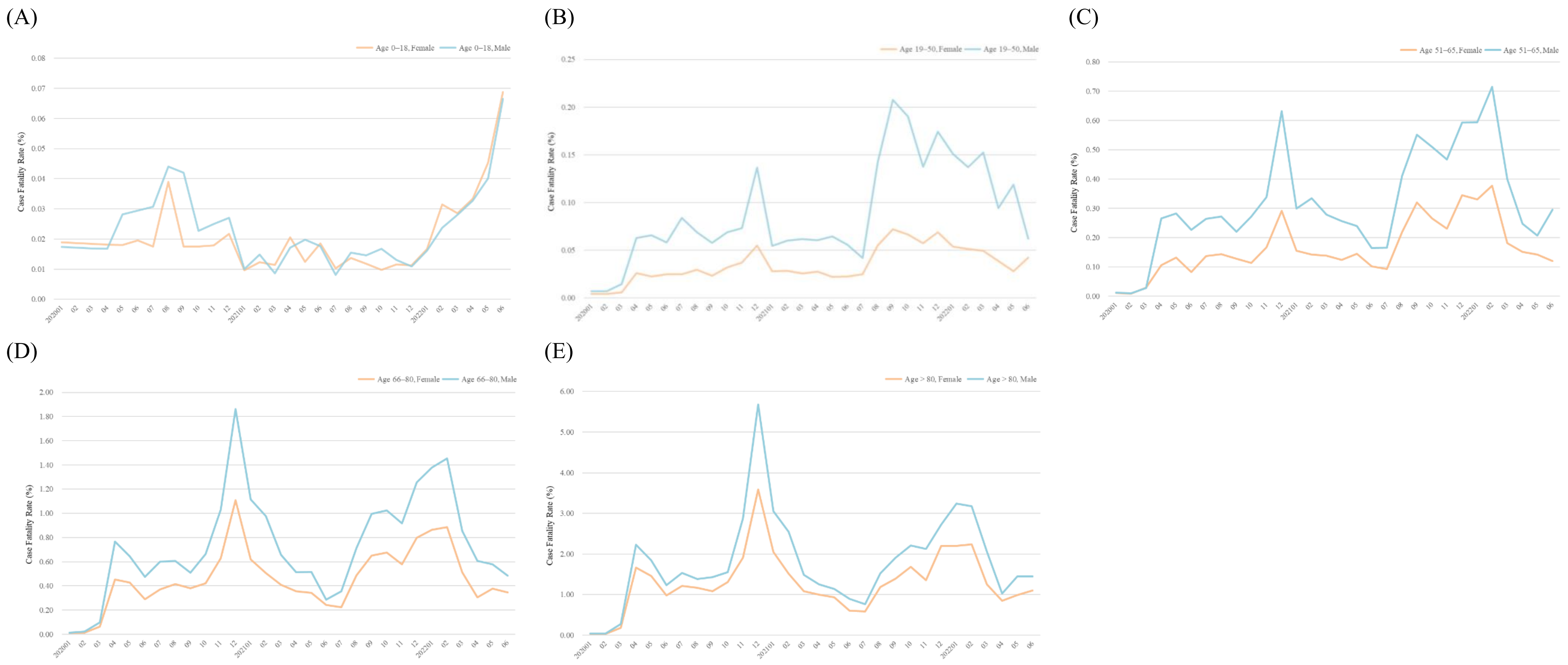

Supplementary Figure 2. Bubble plot of overall case fatality rate (%) versus age groups 0–18, 19–50, 51–65, 66–80 and more than 80. The percentage indicates overall case fatality rate. Number in parenthesis indicates number of deaths during study period. The size of circle is proportional to number of deaths in each age groups.

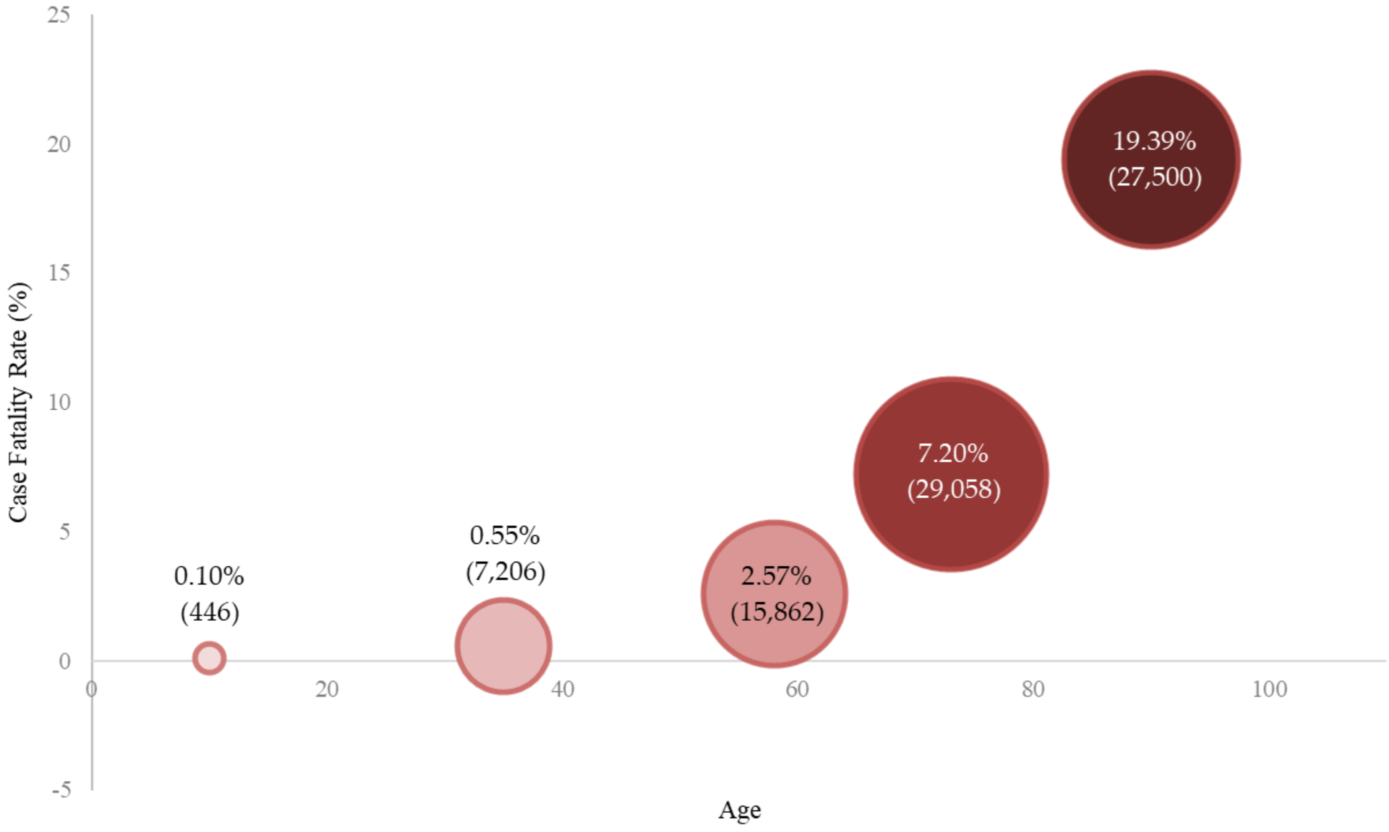

Supplementary Table 1. STROBE statement—checklist of items that should be included in reports of observational studies.

	Item No.	Recommendation	Page No.	Relevant text from manuscript
**Title and abstract**	1	(*a*) Indicate the study's design with a commonly used term in the title or the abstract	Title	
		(*b*) Provide in the abstract an informative and balanced summary of what was done and what was found	Abstract	
**Introduction**				
Background/rationale	2	Explain the scientific background and rationale for the investigation being reported	Introduction, paragraph 1–3	
Objectives	3	State specific objectives, including any prespecified hypotheses	Introduction, paragraph 4	
**Methods**				
Study design	4	Present key elements of study design early in the paper	Methods, paragraph 2.1	
Setting	5	Describe the setting, locations, and relevant dates, including periods of recruitment, exposure, follow-up, and data collection	Methods, paragraph 2.1	
Participants	6	(*a*) *Cohort study*—Give the eligibility criteria, and the sources and methods of selection of participants. Describe methods of follow-up<br>*Case-control study*—Give the eligibility criteria, and the sources and methods of case ascertainment and control selection. Give the rationale for the choice of cases and controls<br>*Cross-sectional study*—Give the eligibility criteria, and the sources and methods of selection of participants	Methods, paragraph 2.2	
		(*b*) *Cohort study*—For matched studies, give matching criteria and number of exposed and unexposed<br>*Case-control study*—For matched studies, give matching criteria and the number of controls per case	Methods, paragraph 2.2	
Variables	7	Clearly define all outcomes, exposures, predictors, potential confounders, and effect modifiers. Give diagnostic criteria, if applicable	Methods, paragraph 2.2	
Data sources/ measurement	8*	For each variable of interest, give sources of data and details of methods of assessment (measurement). Describe comparability of assessment methods if there is more than one group	Methods, paragraph 2.2	
Bias	9	Describe any efforts to address potential sources of bias	Methods, paragraph 2.3	
Study size	10	Explain how the study size was arrived at	NA	
Quantitative variables	11	Explain how quantitative variables were handled in the analyses. If applicable, describe which groupings were chosen and why	Methods, paragraph 2.4	
Statistical methods	12	(*a*) Describe all statistical methods, including those used to control for confounding	Methods, paragraph 2.4	
		(*b*) Describe any methods used to examine subgroups and interactions	Methods, paragraph 2.4	
		(*c*) Explain how missing data were addressed	Methods, paragraph 2.3	
		(*d*) *Cohort study*—If applicable, explain how loss to follow-up was addressed<br>*Case-control study*—If applicable, explain how matching of cases and controls was addressed<br>*Cross-sectional study*—If applicable, describe analytical methods taking account of sampling strategy	Methods, paragraph 2.3	

		(*e*) Describe any sensitivity analyses	NA
**Results**			
Participants	13*	(a) Report numbers of individuals at each stage of study—eg numbers potentially eligible, examined for eligibility, confirmed eligible, included in the study, completing follow-up, and analyzed	Results, paragraph 3.1
		(b) Give reasons for non-participation at each stage	NA
		(c) Consider use of a flow diagram	Figure 6
Descriptive data	14*	(a) Give characteristics of study participants (e.g., demographic, clinical, social) and information on exposures and potential confounders	Results, paragraph 3.1, 3.4
		(b) Indicate number of participants with missing data for each variable of interest	Figure 6
		(c) *Cohort study*—Summaries follow-up time (e.g, average and total amount)	Results, paragraph 3.1
Outcome data	15*	*Cohort study*—Report numbers of outcome events or summary measures over time	Figure 2–7, Table 1
		Case-control study—Report numbers in each exposure category, or summary measures of exposure	NA
		Cross-sectional study—Report numbers of outcome events or summary measures	NA
Main results	16	(*a*) Give unadjusted estimates and, if applicable, confounder-adjusted estimates and their precision (eg, 95% confidence interval). Make clear which confounders were adjusted for and why they were included	Results, paragraph 3.4; Table 1
		(*b*) Report category boundaries when continuous variables were categorized	NA
		(*c*) If relevant, consider translating estimates of relative risk into absolute risk for a meaningful time period	NA
Other analyses	17	Report other analyses done—e.g., analyses of subgroups and interactions, and sensitivity analyses	Results, paragraph 3.2; Table 1–4
**Discussion**			
Key results	18	Summarize key results with reference to study objectives	Discussion, paragraph 4.1~4.5
Limitations	19	Discuss limitations of the study, taking into account sources of potential bias or imprecision. Discuss both direction and magnitude of any potential bias	Discussion, paragraph 4.6
Interpretation	20	Give a cautious overall interpretation of results considering objectives, limitations, multiplicity of analyses, results from similar studies, and other relevant evidence	Discussion, paragraph 4.1~4.5
Generalizability	21	Discuss the generalizability (external validity) of the study results	Discussion, paragraph 4.6
**Other information**			
Funding	22	Give the source of funding and the role of the funders for the present study and, if applicable, for the original study on which the present article is based	page 13

*Give information separately for cases and controls in case-control studies and, if applicable, for exposed and unexposed groups in cohort and cross-sectional studies.

Note: An Explanation and Elaboration article discusses each checklist item and gives methodological background and published examples of transparent reporting. Information on the STROBE Initiative is available at www.strobe-statement.org.

Supplementary Table 2. Search strategy and definition of study group.

Data period: time frame from January 1, 2020 to June 30, 2022

Data access date: April 18, 2023.

Study groups: N= 2,857,925 participants.

**Group 1**				
**Group 1A**				
Must have	any of	laboratory	UMLS:LNC:94534-5	SARS-CoV-2 (COVID-19) RdRp gene [Presence] in Respiratory specimen by NAA with probe detection (labResult: Positive)
		laboratory	UMLS:LNC:94309-2	SARS-CoV-2 (COVID-19) RNA [Presence] in Specimen by NAA with probe detection (labResult: Positive)
		laboratory	UMLS:LNC:94500-6	SARS-CoV-2 (COVID-19) RNA [Presence] in Respiratory specimen by NAA with probe detection (labResult: Positive)
		laboratory	UMLS:LNC:94316-7	SARS-CoV-2 (COVID-19) N gene [Presence] in Specimen by NAA with probe detection (labResult: Positive)
		laboratory	TNX:9088	SARS coronavirus 2 and related RNA [Presence] (labResult: Positive)
		diagnosis	UMLS:ICD10CM:J12.82	Pneumonia due to coronavirus disease 2019
		diagnosis	UMLS:ICD10CM:U09.9	Post COVID-19 condition, unspecified
		diagnosis	UMLS:ICD10CM:U09	Post COVID-19 condition
		diagnosis	UMLS:ICD10CM:U07.1	COVID-19
		diagnosis	UMLS:ICD10CM:U07.2	COVID-19, virus not identified (WHO)
	and	visit	TNX:Visit	Visit
Date constraint		The terms in this group occurred between Jan 1, 2020 and June 30, 2022		
Event relationship		Any instance of Group 1B occurred at least 1 day before the first instance of Group 1A		
**Group 1B**				
Cannot have		demographics	Deceased	Deceased

Comorbidities and Medications:

**Codes**	**Comorbidities/Medications**
ICD-10-CM code	
I10–I16	Hypertensive diseases
I60–I69, G45	Cerebrovascular diseases
N18	Chronic kidney disease
J40–J47	Chronic respiratory diseases
E10–E14	Diabetes mellitus
F03	Dementia
E78	Dyslipidemia

I20–I25	Ischemic heart diseases
K70–K77	Liver diseases
C00–C97	Malignancy
E65–E68	Overweight and obesity
F17	Smoking
ATC code	
C09	ACEi/ARB
C07	Beta-blocking agents
C08	Calcium channel blockers
A10BA02	Metformin
C10	Lipid modifying agents
C05AA	Corticosteroids
M01A	Non-steroidal anti-inflammatory drugs
N05A	Antipsychotics

ACEis, angiotensin converting enzyme inhibitors; ARBs, angiotensin II receptor blockers; ATC, Anatomical Therapeutic Chemical; ICD-10-CM, International Classification of Diseases, Tenth Revision, Clinical Modification.

Supplementary Table 3. Variants of concern in the United States.

WHO label	PANGO lineage	Country first detected	Year and month of first detected	Year and month of first identified in United States	Proportion (%) during the third surge	Proportion (%) during the fourth surge
Currently circulating VOCs						
Omicron	B.1.1.529	Botswana	November 2021	December 2021	0	97.03
Omicron subvariants						
BA.1	B.1.1.529.1	Botswana	November 2021	December 2021	NA	NA
BA.2	B.1.1.529.2	South Africa	November 2021	January 2022	NA	NA
BA.3	B.1.1.529.3	South Africa	November 2021	NA	NA	NA
BA.4	B.1.1.529.4	South Africa	January 2022	April 2022	NA	NA
BA.5	B.1.1.529.5	South Africa	January 2022	May 2022	NA	NA
De-escalated VOCs						
Alpha	B.1.1.7	United Kingdom	September 2020	December 2020	0.37	0
Beta	B.1.351	South Africa	May 2020	June 2021	<0.01	0
Gamma	P.1	Brazil	November 2020	January 2021	0	0
Delta	B.1.617.2	India	October 2020	February 2021	98.69	2.91

PANGO, Phylogenetic Assignment of Named Global Outbreak Lineages; VOC, variant of concern; WHO, World Health Organization.

Assess date on July 23, 2022, to COVID Data Tracker.

NA, not available.

Supplementary Table 4. Impact of COVID-19 variants on transmissibility, hospital admission, mortality, and vaccination efficacy profile against variants.

WHO label	PANGO lineage	Transmissibility	Hospitalization	Mortality	Vaccination efficacy	Immune escape
Omicron	B.1.1.529	2.00 (1.75–2.40) [1]<br>1.37 (0.21–0.60) [1]<br>relative to Delta	0.41 (0.39–0.43) [2]<br>relative to Delta<br>0.21 (0.19–0.23) [3]<br>relative to Alpha	0.31 (0.26–0.37) [2]<br>relative to Delta<br>0.15 (0.11–0.21) [3]<br>relative to Alpha	0.22 (0.20–0.24) [2]‡ relative to unvaccinated, booster dose<br>0.65 (0.51–0.75) [4]‡, 2 doses<br>0.86 (0.77–0.91) [4]‡, 3 doses<br>0.40 (0.26–0.51) [5]§, 2 doses	++++
Alpha	B.1.1.7	1.29 (1.24–1.33) [6]	1.52 (1.47–1.57) [7]	1.59 (1.44–1.74) [7]<br>1.67 (1.34–2.09) [8]	0.85 (0.82–0.88) [4]‡, 2 doses<br>0.92 (0.90–0.93) [9]§, 2 doses<br>0.73 (0.66–0.78) [9]†, 2 doses<br>0.91 (0.89–0.93) [5]§, 2 doses	+
Beta	B.1.351	1.25 (1.20–1.30) [6]	No evidence	Possibly increased [10]	Efficacy reduction	++++
Gamma	P.1	1.38 (1.29–1.48) [6]	Possibly increased [11]	Possibly increased [11]	Retained	++
Delta	B.1.617.2	1.97 (1.76–2.17) [6]	1.85 (1.39–2.47) [9]<br>relative to Alpha<br>1.12 (0.93–1.15) [12]	1.14 (0.50–2.23) [12]	0.85 (0.83–0.87) [4]‡, 2 doses<br>0.94 (0.92–0.95) [4]‡, 3 doses<br>0.79 (0.75–0.82) [9]§, 2 doses<br>0.60 (0.53–0.66) [9]†, 2 doses<br>0.82 (0.75–0.87) [5]§, 2 doses	++

PANGO, Phylogenetic Assignment of Named Global Outbreak Lineages; WHO, World Health Organization.

§Pfizer–BioNTech BNT162b2, †Oxford–AstraZeneca ChAdOx1, ‡mRNA vaccines

+: Resistance to partial monoclonal antibodies (mAbs)

++: Resistance to partial mAbs, convalescent plasma and partial vaccine.

++++: Resistance even immune escape to many mAbs, convalescent plasma and many vaccines.

Reference:

1. Yang, W. and J. Shaman, *COVID-19 pandemic dynamics in South Africa and epidemiological characteristics of three variants of concern (Beta, Delta, and Omicron).* medRxiv, 2022.
2. Nyberg, T., et al., *Comparative analysis of the risks of hospitalisation and death associated with SARS-CoV-2 omicron (B.1.1.529) and delta (B.1.617.2) variants in England: a cohort study.* Lancet, 2022. **399**(10332): p. 1303-1312.
3. Christensen, P.A., et al., *Signals of Significantly Increased Vaccine Breakthrough, Decreased Hospitalization Rates, and Less Severe Disease in Patients with Coronavirus Disease 2019 Caused by the Omicron Variant of Severe Acute Respiratory Syndrome Coronavirus 2 in Houston, Texas.* Am J Pathol, 2022. **192**(4): p. 642-652.
4. Lauring, A.S., et al., *Clinical Severity and mRNA Vaccine Effectiveness for Omicron, Delta, and Alpha SARS-CoV-2 Variants in the United States: A Prospective Observational Study.* medRxiv, 2022.
5. Katrine, G.M.A.E.H.-D.S.A.B.F.N.U.N., *Vaccine effectiveness against SARS-CoV-2 infection and COVID-19-related hospitalization with the Alpha, Delta and Omicron SARS-CoV-2 variants: a nationwide Danish cohort study.* Cold Spring Harbor Laboratory, 2022.
6. Campbell, F., et al., *Increased transmissibility and global spread of SARS-CoV-2 variants of concern as at June 2021.* Euro Surveill, 2021. **26**(24).
7. Nyberg, T., et al., *Risk of hospital admission for patients with SARS-CoV-2 variant B.1.1.7: cohort analysis.* BMJ, 2021. **373**: p. n1412.
8. Grint, D.J., et al., *Case fatality risk of the SARS-CoV-2 variant of concern B.1.1.7 in England, 16 November to 5 February.* Euro Surveill, 2021. **26**(11).
9. Sheikh, A., et al., *SARS-CoV-2 Delta VOC in Scotland: demographics, risk of hospital admission, and vaccine effectiveness.* Lancet, 2021. **397**(10293): p. 2461-2462.
10. Organization, W.H. *Weekly epidemiological update on COVID-19 - 20 July 2021*. 2021; Available from: https://www.who.int/publications/m/item/weekly-epidemiological-update-on-covid-19---20-july-2021.
11. Nonaka, C.K.V., et al., *SARS-CoV-2 variant of concern P.1 (Gamma) infection in young and middle-aged patients admitted to the intensive care units of a single hospital in Salvador, Northeast Brazil, February 2021.* Int J Infect Dis, 2021. **111**: p. 47-54.
12. R., F.D.N.T.A., *Progressive Increase in Virulence of Novel SARS-CoV-2 Variants in Ontario, Canada.* Cold Spring Harbor Laboratory, 2021.
